# Nanoscale photobiotinylation, pulldown and sequencing of region-specific DNA from intact cells

**DOI:** 10.1101/2023.12.04.569951

**Published:** 2023-12-06

**Authors:** Thomas C. Roberts, Max Kushner, Jack C. Crowley, Abdullah Ozer, Juan Wang, Judhajeet Ray, John T. Lis, Warren R. Zipfel

**Affiliations:** 1 Smith School of Chemical and Biomolecular Engineering, Cornell University, Ithaca, NY USA 14853; 2 Field of Biophysics, Cornell University, Ithaca NY USA 14853; 3 Applied and Engineering Physics, Cornell University, Ithaca, NY USA 14853; 4 Department of Molecular Biology and Genetics, Cornell University, Ithaca NY USA 14853; 5 Meinig School of Biomedical Engineering, Cornell University, Ithaca NY USA 14853

## Abstract

Femto-seq is a novel nanoscale optical method that can be used to obtain DNA sequence information from targeted regions around a specific locus or other nuclear regions of interest. Two-photon excitation is used to photobiotinylate femtoliter volumes of chromatin within the nucleus, allowing for subsequent isolation and sequencing of DNA, and bioinformatic mapping of any nuclear region of interest in a select set of cells from a heterogenous population.

Spatio-temporal changes in nuclear chromatin organization are fundamental to understanding how the nucleus functions. The detailed structural organization of the genome has become clearer over the past few decades with the development of high-resolution microscopy coupled with multiplexed FISH and in situ genome sequencing, sequencing-based methods such as Hi-C and its derivatives, and new computational approaches^1^.

Here we present a new approach designed to look at the genomic sequences located within a region of interest from user-selected cells of interest. The method, called Femto-seq (Figure 1), combines 3D localized two-photon excitation dependent photo-biotinylation in targeted nuclear volumes as small as a cubic micron (femtoliter) followed by chromatin isolation and DNA sequencing. The nuclear volumes from which sequence information can be obtained can be as small as the two-photon point spread function (PSF) of the microscope (Fig. 1D–F). Upon light membrane permeabilization, our optimized photoactivatable DNA crosslinker with a biotin affinity tag^2^ (AP3B – Fig 1A) enters cells and intercalates into DNA. Using confocal microscopy, the fluorescently labeled regions of interest (ROIs) are located and two-photon excitation at 700 nm is used to photoactivate the crosslinker within these regions (Fig. 1G). ROIs in the nucleus are assigned based on 3D confocal imaging of fluorescence markers. ROIs can be labeled gene loci, nuclear bodies or other distinguishable structures, and since identification is imaging based, the user can select which cells are targeted. For example, a sub-set may be targeted based on cell state or expression of a specific marker. After crosslinking, cells are washed to remove non-crosslinked AP3B. Chromatin is extracted from the pre-washed cells, and then sheared and purified using the biotin tag. After crosslink reversal and adapter ligation, the captured chromatin fragments are sequenced. Sequencing data can provide information about the spatial association between regulatory sequences and the targeted gene loci, or sequences associated with certain sub-nuclear compartments.

Figure 1 shows an overview of the method and the possible photo-biotinylation volumes (Fig. 1A–F). We validated Femto-seq with a transgenic U2OS 2-6-3 cell line (transgene shown in Figure 1H) provided by the Spector Laboratory^3^. The cells were further modified by stable co-transfection with pSV2-EYFP-LacI plasmid to express LacI-YFP, and the transgene was located by imaging the LacO/LacI-YFP spots. Cells are maintained in the presence of IPTG until an hour before use to block cell division inhibition by LacI binding to the LacO repeats. The cell line also allows for Tet/Dox inducible transcriptional activation, which can be monitored by CFP expression and/or MCP-RFP localization. Based on initial experiments to determine expected losses during chromatin pull-down and isolation, and the small size of the nuclear volume around the transgene locus being irradiated, we estimated ~15,000 cells per experiment would be required for obtaining sufficient photobiotinylated DNA for sequencing. Cells were cultured on a coverglass-bottom dish and one hour prior to imaging incubated with the photobiotinylation reagent. The imaging and photoactivation were carried out using a Zeiss LSM 880 multiphoton/confocal microscope. YFP labeled loci were identified by confocal imaging. We created a macro on the LSM 880 to transfer image data to a lab-written dynamic link library (DLL) that found the transgene loci and returned the corresponding ROIs to the Zeiss software. We used the photobleaching module to irradiate the ROIs with 700 nm femtosecond pulses delivered through a 0.5 NA air objective lens. We irradiated a 4x4 pixel box (3.32 x 3.32 μm) centered on the activated transgene. The extent of the axial 2P excitation was ~2.5 microns, and we estimate the photo-biotinylated volume targeted around the transgene locus to be ~28 μm^3^. After irradiation of the ROIs, cells were incubated for one hour in 1,6-hexanediol to remove non-covalently linked AP3B from nuclei. To account for bias associated with AP3B chromatin accessibility, ~15,000 cells were incubated with AP3B as described above and uniformly irradiated by UV light (365 nm) in a StrataLinker 2400 (Stratagene) UV box. Biotinylated DNA from the UV irradiated cells was isolated, sheared, captured with Streptavidin beads, and sequenced and used to normalize coverage. Measurements of U2OS nuclear volume in our cells yielded a value of ~600 μm^3^ (similar to a value of ~700 μm^3^ reported in ref. 4). Assuming spatially homogenous chromatin, we would expect an enrichment factor of ~21 (600/28). After a 1,6-hexanediol wash step to remove non-covalently bound AP3B, purification of the biotinylated DNA fraction and sequencing, a 14.8-fold enrichment of the targeted transgene sequences was found over untargeted rDNA background sequences (Fig. 2A and B) after normalizing crosslinking to the uniformly UV irradiated sample. Given the assumptions made above, this is in reasonable agreement with the predicted enrichment.

Interestingly, a genome-wide analysis found that both chromosomes 1 and 18 had significant enrichment. Coverage plots showed the enrichment of Chr1 was around a 10 MB region of the p36 arm, consistent with the reported insertion site^3^ (Fig. 2C). To explore the appearance of the Chr18 enrichment in our Femto-seq results we performed FISH experiments targeting Chr1 and Chr18 to visualize proximity to the transgene. Metaphase chromosomes (Fig. 2E–I) confirmed that a translocation between the tip of Chr1 where the transgene was originally inserted and Chr18 had occurred, explaining the chromosome-wide enrichment of Chr18 (Fig. 2D), in addition to the expected Chr1 p36 arm sub-chromosomal enrichment.

In our validation experiments we used lightly-permeabilized live cells, but Femto-seq can also be applied to cells prepared for DNA-FISH enabling any gene of interest to be targeted. AP3B will intercalate and can be photo-crosslinked in cells fixed and prepared for FISH, as shown in Figure 1E and in the Supplementary Information section (Figure S1). DNA can also be harvested from these cells as prepared under most FISH protocols (see SI, Figure S2).

Using Femto-seq we can isolate DNA for sequencing from any volume of interest in the nucleus that can be located by high resolution of imaging, for example, to uncover sequence-specific relationships that may exist around nuclear bodies or near active super-enhancers. Because individual cells are imaged, Femto-seq also allows for specific phenotypes within a population to be exclusively targeted. In summary, our method is a unique new tool for analyzing 3D genomic architecture that sits in a unique space between imaging-based methods and chromosome capture techniques.

## Supplementary Material

1

## Figures and Tables

**Figure 1.**
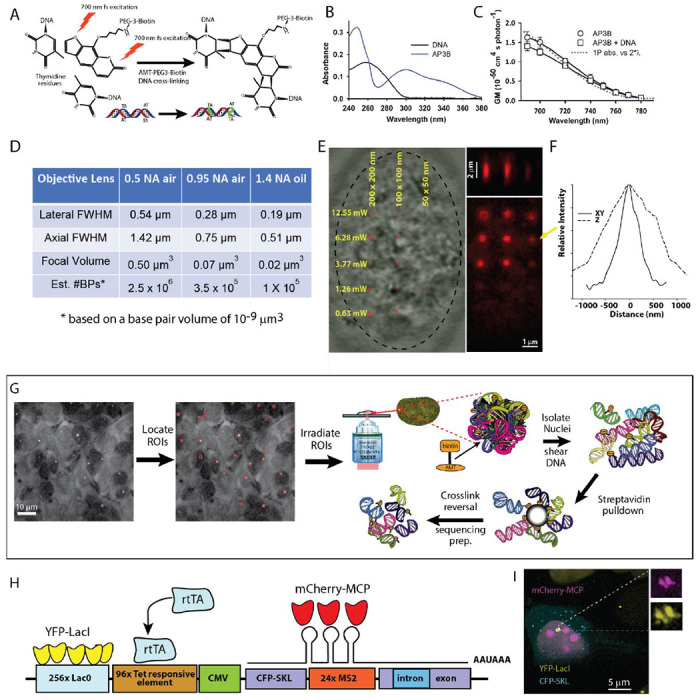
Targeted nanoscale photobiotinylation using Femto-seq. **A**. 4’-aminomethyltrioxsalen (4’-AMT) is a psoralen family DNA cross-linker, which forms covalent bonds primarily with thymidine residues when excited by UV or UV-like two-photon (2P) irradiation. The photoactivatable reagent used is 4′-Aminomethyltrioxsalen-PEG3-Biotin (AP3B), which enables covalent attachment of biotin to DNA. **B.** Absorbance spectra of AP3B. Use of 700 nm 2P excitation or longer UV (~360 nm) avoids shorter wavelength DNA absorptions. **C.** Two-photon absorption cross-sections of the AP3B photobiotinylation probe. **D.** The minimal volume (assuming a single XYZ spot) calculated for three different numerical aperture (NA) objectives. Using high NA, it is possible to biotinylate extremely small volumes within the nucleus. **E**. Streptavidin-Alexa 647 labeling of different sized photo-biotinylated regions (1x1, 2x2 and 4x4 pixel ROI box sizes) in a FISH-fixed U2OS nucleus after photo-biotinylation taken at 5 different cross-linking intensities. The 700 nm femtosecond pulses were delivered through 63x/1.4 Zeiss objective lens. Pixel size was 50 nm (5.3x zoom) and the pixel dwell time as 4.1μs and 30 passes over the ROI was used (123 μs per 0.0025 μm^3^) for all crosslinking powers shown. **F.** Lateral and axial dimensions a one-pixel region cross-linked using 6.28 mW at the above scanning parameters. **G.** Overview of the Femto-seq method (see text). **H.** U2OS transgene structure (adapted from ref. 3) used for Femto-seq validation experiments. **I.** U2OS cell after gene activation (+DOX) and IPTG removal to allow YFP-LacI binding for visualization of transgene site showing colocalization of the YFP-LacI and MCP-mCherry.

**Figure 2.**
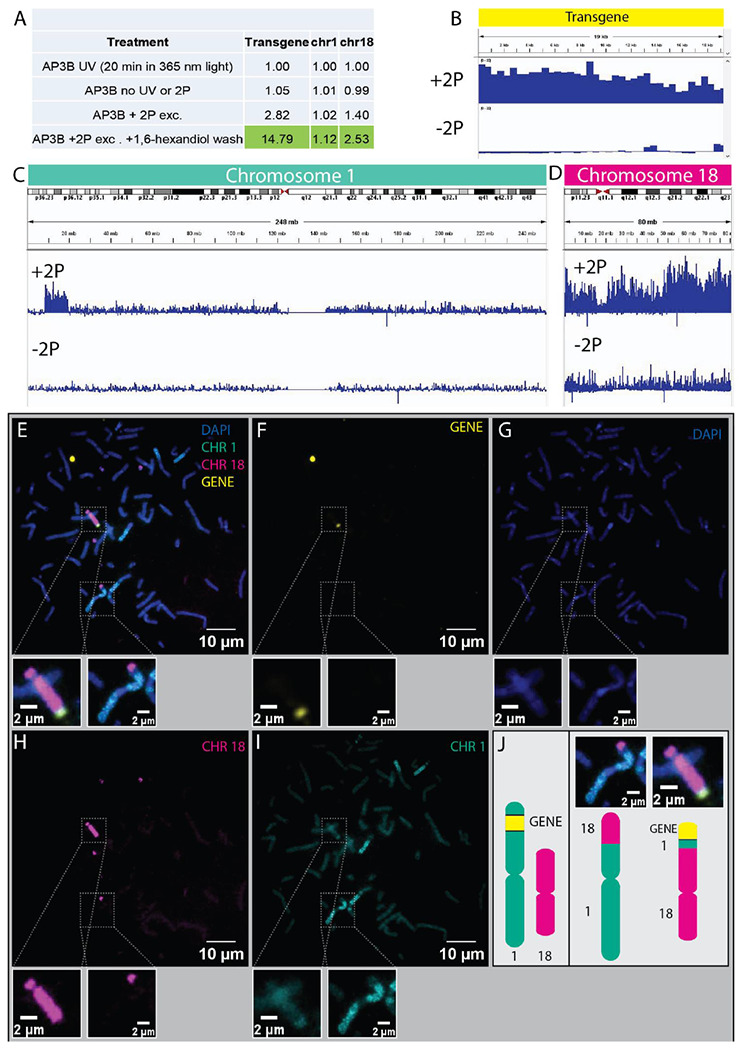
Targeted photo-biotinylation and isolation of chromatin from a transgene locus in cultured U2OS cells. **A**. Calculated enrichments of selected genomic regions from different treatments (normalized to uniform UV irradiation reads). **B-D**. Browser shots of transgene, Chromosome 1, and Chromosome 18, respectively, mapping read coverage as the ratio of targeted 2P-treated to UV-treated to control for non-uniform AP3B selectivity. (Unmapped reads in C are q12 peri-centromeric region.) **E-I**. Metaphase DNA-FISH on U2OS cells. Transgene locus in yellow, Chr1 in green, Chr18 in magenta, DAPI in blue. **J**. Schematic of the presumed transgene location from ref 3 (left) and the translocated transgene (right) which we determined initially with Femto-Seq, and then subsequently verified with multicolor FISH.
