# Supplementary material for: Nanoscale photobiotinylation, pulldown and sequencing of region-specific DNA from intact cells
Source: bioRxiv. 2023 Dec 6:2023.12.04.569951. Preprint. [Version 1] doi: 10.1101/2023.12.04.569951 (PMC10723339; doi:10.1101/2023.12.04.569951)
Supplement: 1 [file NIHPP2023.12.04.569951v1-supplement-1.pdf]

bioRxiv preprint doi: https://doi.org/10.1101/2023.12.04.569951; this version posted December 6, 2023. The copyright holder for this preprint (which was not certified by peer review) is the author/funder, who has granted bioRxiv a license to display the preprint in perpetuity. It is made available under aCC-BY 4.0 International license.

Online methods section.

Photo-biotinylation reagent. Femto-seq utilizes a specifically designed photoactivatable DNA crosslinker with an affinity tag, 4′-Aminomethyltrioxsalen-PEG3-Biotin (AP3B). The photoactivatable component is a psoralen derivative, 4'-aminomethyltrioxsalen (4'-AMT). The compound was synthesized as described in ref 1. The compound has a polyethylene glycol trimer linker to increase solubility. The 3-PEG is terminated with a biotin moiety, which is used as the affinity tag. AP3B is similar to the commercially available EZ-link psoralen-PEG3-biotin probe (ThermoFisher # 29986), but we have found AP3B to be a more efficient photo-crosslinking reagent.

Cell line modifications. A modified clone of the human bone osteosarcoma epithelial U2OS 2-6-3 cell-line[2] was gifted by the Spector Laboratory at Cold Spring Harbor Laboratory. The cells feature ~200 copies of a transgenic construct, each containing 256 Lac Operator (LacO) repeats, 96 tetracycline-responsive elements which regulate a minimal CMV promoter, and an open reading frame. Transcription of the coding sequences produce CFP-tagged SKL proteins, as well as mRNA containing 24 MS2 sequences when activated. We modified the cell line to stably expresses LacI-YFP to locate the transgene locus by confocal microscopy by accumulation of LacI-YFP at the LacO repeats, and rtTA to enable transcription by the addition of doxycycline. This was accomplished by co-transfection with pSV2-EYFP-LacI plasmid and pCDH puromycin resistant plasmid at a ratio of 1:20. 5mM Isopropyl β-D-1-thiogalactopyranoside (IPTG, Sigma-Aldrich I5502) was added to the media to allow cell division, since binding of LacI-YFP to the LacO site will block DNA replication. IPTG was replenished every 2-3 days. 1 μg/ml puromycin was added to the select successfully transfected cells. To add the rtTA construct, U2OS 2-6-3 LacI-EYFP cells were co-transfected with pTetOn plasmid and pCDH blasticidin resistant plasmid at a ratio of 1:20. 15 μg/ml blasticidin was added to the select successfully transfected cells. When the distinct single colonies were formed (2-3 weeks after selection started), 72 were selected and

bioRxiv preprint doi: https://doi.org/10.1101/2023.12.04.569951; this version posted December 6, 2023. The copyright holder for this preprint (which was not certified by peer review) is the author/funder, who has granted bioRxiv a license to display the preprint in perpetuity. It is made available under aCC-BY 4.0 International license.

plated onto two 48-well plates (with puromycin and IPTG). 50 cells survived and were plated on MatTek dishes for imaging. We found 9 that showed CFP-SKL expression after adding doxycycline, and 7 monoclonal cell-lines showed bright yellow spots after IPTG removal. The monoclonal cell-lines that showed bright yellow spots in the nuclei and CFP-SKL expression in the cytosol were frozen for later use.

Cell culture. U2OS 2-6-3 cells were kept in a 37°C incubator in Dulbecco's modified Eagle's medium (DMEM) containing 10% fetal bovine serum (FBS) and 1% antibiotic-antimycotic (Gibco). The media, also containing 5µM of IPTG, was replenished every 2 days. Cells were cultured on tissue culture-treated plastic at 37°C and 5% CO_2, and maintained at 10-80% monolayer confluency.

Cell sample preparation for Femto-seq. 10,000 U2OS 2-6-3 cells stably expressing LacI-YFP and pTetOn were plated at the center of a #1.5 coverslip-bottomed (7 mm dia.) MatTek dish in a 50 µL volume. After allowing the cells to settle on the surface of the plate, 2 mL of DMEM containing 5 mM IPTG and 5 µg/mL doxycycline was added to the dish. Cells were then incubated overnight at 37°C. The following day, 6 hours before irradiation, media was removed and 2 mL of fresh DMEM media without IPTG was added. One hour before irradiation cells were permeabilized by incubation in 0.2% Triton-X-100 in a stabilizing buffer (4% PEG 8000, 1mM EGTA, 10mM PIPES) for 5 minutes. Cells were then washed three times with 1 mL PBS and then incubated in 200 µM AP3B in PBS for 1 hour at 37°C.

Confocal Imaging, ROI targeting and microscope automation. For the transgene validation experiments reported here, LacI-YFP loci were visualized using a 20x/0.5NA objective lens and 514 nm excitation. Once a field of view was imaged, a Zeiss Zen VBA macro is run which sends the image data to a dynamic link library (DLL) for analysis. The DLL finds the fluorescent loci and exports a text file of the rectangular ROIs around each locus back into the Zen macro. The loci (fluorescent spots) are found using the algorithm described in ref. 3. The user then uses the photobleaching module to irradiate the

bioRxiv preprint doi: https://doi.org/10.1101/2023.12.04.569951; this version posted December 6, 2023. The copyright holder for this preprint (which was not certified by peer review) is the author/funder, who has granted bioRxiv a license to display the preprint in perpetuity. It is made available under aCC-BY 4.0 International license.

ROIs with 700 nm two-photon excitation to photobiotinylate the DNA. The macro and DLL are available from the corresponding author.

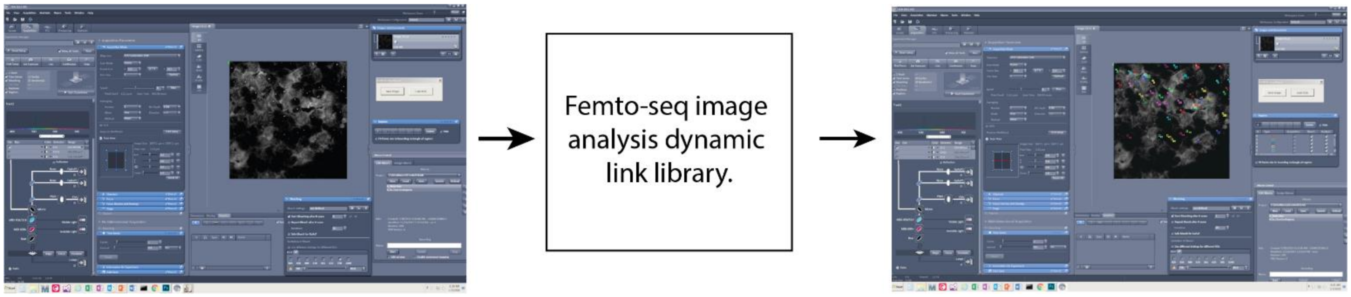

Figure S1. Use of Zeiss LSM 880 confocal/multiphoton system for Femto-seq. A VBA (Visual Basic for Applications) macro written for the Zeiss Zen software (version 2.3 SP1) sends the image data (2D array of bytes - typically 512 x 512) to a dynamic link library (Femtoseq.dll) written in C/C++ (Visual Studio 2019) which locates the fluorescent loci and generates ROIs. The ROIs are imported back into Zen and laser irradiation of the regions using 700 nm femtosecond laser pulses is carried out using the ZEN photobleaching module.

In the validation experiment presented here, 4x4 pixel boxes centered on the LacI-YFP locus were used to specify the regions to irradiate. After a field of view was irradiated, the stage was moved to a new field of cells and the process repeated. Around 5000 cells could be irradiated per dish before the YFP contrast was lost (due to LacI-YFP disassociation). In these experiments 30 passes over each ROI was used. Laser power was ~70 mW delivered through a 0.5 NA objective lens, with a pixel dwell time of 6.3μs and pixel size of 50 x 50 nm. The power we used to activate the AP3B cross-linker and biotinylate the DNA in the transgene experiments was higher than, for example, the powers used in the power series shown in Figure 1E. Adjusting for the differing numerical apertures (1.4 in Figure 1E vs 0.5 in our transgene experiments) and based on the measured two-photon absorption cross-section at 700 nm (~1.5 GM), the number of excitations per pixel dwell time at the center of the point spread function would be ~50 at 0.5 NA and 70 mW, compared to ~70 using a 1.4 NA objective at 12.5 mW (i.e. as in Figure 1E). For our transgene validation experiments, we used higher powers both due to the lower NA and the fact that we

bioRxiv preprint doi: https://doi.org/10.1101/2023.12.04.569951; this version posted December 6, 2023. The copyright holder for this preprint (which was not certified by peer review) is the author/funder, who has granted bioRxiv a license to display the preprint in perpetuity. It is made available under aCC-BY 4.0 International license.

do not know the two-photon *cross-linking* quantum yield. We also were trying to capture a larger volume around the transgene site, so the higher power produced a greater crosslinking depth (i.e. as in Fig. 1E).

Initial optimization experiments.

As we developed the Femto-seq methodology, we carried out a number of initial experiments using the U2OS transgene cells that led to the successful demonstration experiment shown here. These involved different laser powers and ROIs doses, and various "tweaks" to the isolation procedure which we will not cover in detail, with the exception of noting two areas which we have found to be critical. The first is the need for accurate 3D co-alignment of the femtosecond laser focus with the confocal excitation used to locate the target regions. The second is the use of 1,6-hexanediol to help remove non-covalently bound AP3B from the chromatin before streptavidin pulldown. The latter reduced the non-targeted background DNA sequences by more than a factor of 5 (Fig. 2A).

Femto-seq Library Preparation for Illumina Sequencing

Following irradiation, cells were washed with 1,6-hexanediol (50mM) for 1 hour on the dish. The cells were then detached from the dish with 0.05% Trypsin-EDTA (Gibco) and spun down and stored at -80°C until DNA extraction. Genomic DNA from cross-linked (2P or UV) and non-cross-linked cells were extracted and fragmented to 200-800 bp by sonication. Biotinylated fragments are then captured by Dynabeads MyOne C1 streptavidin beads, and while on beads, end repair, A-tailing, and TRUseq adapter ligation was performed. Adapter ligated fragments are then eluted, and psoralen cross-links chemically reversed in the presence of 3M Urea, 0.1 M KOH, 1 mM EDTA at 90°C. The resulting library is PCR amplified and submitted for paired-end Illumina sequencing (2x 37bp) on a HiSeq 2000 or a NextSeq 500 instrument.

The resulting sequencing data is processed using a custom script. Briefly, paired-end reads are mapped to a custom human genome where the transgene plasmid sequences are included as a separate chromosome

bioRxiv preprint doi: https://doi.org/10.1101/2023.12.04.569951; this version posted December 6, 2023. The copyright holder for this preprint (which was not certified by peer review) is the author/funder, who has granted bioRxiv a license to display the preprint in perpetuity. It is made available under aCC-BY 4.0 International license.

using bowtie2 software[4]. Mapped reads were visualized using IGV Genome Browser and reads that mapped to regions of interest were counted using bedtools[5] and deeptools software[6].

Femto-seq Library Preparation Protocol

A. DNA isolation.

1. 2P- or UV-crosslinked cells (15-30K cells in 300 µl 1xPBS) were lysed with equal volume of 2x Lysis Buffer (200 mM Tris-Cl pH 8.5, 200 mM EDTA, 2% SDS).
2. Incubate at 70°C for 30 min.
3. Cool down to room temperature (~15 min).
4. Add 5 µl of RNase A/T1 cocktail per sample.
5. Incubate at 37°C for 30 min.
6. Add 86 µl of 8M KAc (final concentration 1M). Mix gently.
7. Incubate on ice for 30 min.
8. Centrifuge at room temperature max speed for 15 min to pellet proteins and cellular debris.
9. Transfer supernatant to a new tube.
10. Extract DNA with equal volume Phenol/Chloroform (1:1) mix. Mix thoroughly but gently.
11. Centrifuge at room temperature max speed for 5 min.
12. Transfer upper aqueous phase to a new tube.
13. Repeat Phenol/Chloroform extraction once more.
14. Extract DNA with equal volume Chloroform. Mix thoroughly but gently.
15. Centrifuge at room temperature max speed for 3 min.
16. Transfer upper aqueous phase to a new tube.
17. To precipitate genomic DNA (~600 µl), add 2 µl of GlycoBlue co-precipitant, 1/10 volume 3M NaAc pH 5.2, and equal volume of isopropanol.
18. Incubate at room temperature for 30 min.
19. Centrifuge at 4°C max speed for 30 min. Discard supernatant after centrifuge.
20. Rinse the pellet with 750 µl 70% EtOH.
21. Centrifuge at 4°C max speed for 5 min. Discard supernatant after centrifuge.
22. Air dry pellet for ~5 min.
23. Resuspend each pellet in 200 µl of 1x TE (10 mM Tris-Cl pH 7.5 + 0.1 mM EDTA)
24. Add 1 µl of RNase A/T1 cocktail and incubate at 37°C for 30 min.
25. Fragment the genomic DNA to 200-800 bp by sonication using Diagenode Bioruptor (Denville, NJ) at low setting, 30 sec ON/60 sec OFF for 15 min with ice-cold water in the reservoir.
26. Store DNA at -20°C.

B. Biotinylated DNA Pull-down with Streptavidin beads:

1. Thaw fragmented genomic DNA on ice.

bioRxiv preprint doi: https://doi.org/10.1101/2023.12.04.569951; this version posted December 6, 2023. The copyright holder for this preprint (which was not certified by peer review) is the author/funder, who has granted bioRxiv a license to display the preprint in perpetuity. It is made available under aCC-BY 4.0 International license.

2. Resuspend Dynabeads MyOne Streptavidin C1 beads by vortexing.
3. Transfer 40 µl of bead slurry per sample to a clean tube.
4. Wash the beads 3 times with 5 volumes of TWB buffer (0.05% Tween-20, 5mM Tris-Cl pH 8.0, 0.5 mM EDTA, 1M NaCl). Use magnet to collect beads on the side of the tube and remove the buffer carefully with a pipette.
5. Resuspend beads with 5 volumes of 2X BWB buffer (10mM Tris-Cl pH 8.0, 1mM EDTA, 2M NaCl).
6. Add equal volume (200 µl) of pre-washed beads to each sample.
7. Incubate at room temperature for 30 min on a rotator (or Thermomixer @600 rpm).
8. After binding wash beads, twice with 300 µl of 1x BWB buffer, and twice with 300 µl TWB buffer.
9. For each wash, collect the beads on the side of the tube using a magnet for 2 minutes, remove and discard the buffer by pipetting. Transfer beads with the new wash buffer to a new tube each time.

C. DNA End Repair on Beads:

1. Resuspend beads in End Repair mix:

	Per sample
DNA on beads	
10X T4 DNA Ligase Buffer (NEB)	5 µl
2.5 mM dNTP mix	5 µl
T4 DNA Polymerase	2 µl
T4 PNK	2 µl
Klenow Polymerase	0.5 µl
ddH2O	35.5 µl

2. Incubate at RT Thermomixer 600 rpm for 30 min.
3. Wash beads 2x 0.4 ml 1x BWB buffer and once with 0.4 ml 1x NEB Buffer 2.
4. Resuspend beads in A-tailing master mix:

	Per sample
DNA on beads	
10X NEB Buffer 2	5 µl
1.0 mM dATP	10 µl
Klenow exo-polymerase	2 µl
ddH2O	33 µl

5. Incubate at 37C Thermomixer 600 rpm for 30 min.
6. Wash beads 2x 0.4ml 1x BWB buffer and once with 0.4 ml 1x homemade T4 DNA Ligase buffer (-ATP).
7. Resuspend beads in 50 µl of 1.1X T4 DNA Ligase Buffer (NEB, with ATP).
8. Add 2 µl of 0.3 uM Indexed TRUseq adapter + 3 µl of T4 DNA Ligase (NEB, 1200 units).
9. Incubate ligation reaction at 16°C overnight.
10. Next day, wash beads 2x 0.4ml 1x TWB buffer and once with 0.4 ml BWB buffer. Transfer beads to a clean tube with each wash.

bioRxiv preprint doi: https://doi.org/10.1101/2023.12.04.569951; this version posted December 6, 2023. The copyright holder for this preprint (which was not certified by peer review) is the author/funder, who has granted bioRxiv a license to display the preprint in perpetuity. It is made available under aCC-BY 4.0 International license.

11. Elute DNA from beads with 56 µl Elution Buffer (10 mM EDTA, 95% Formamide). Incubate at 65°C for 10 minutes.
12. Collect beads on magnet and transfer supernatant to a new tube.
13. Collect residual beads on magnet and transfer supernatant to a new tube again. This step is critical - if any residual magnetic beads are left in the elution it will cause DNA degradation during the next crosslink reversal step.

D. Psoralen crosslink reversal and library prep:

Crosslink reversal buffer:

	Final conc	1x
DNA Eluate		56 µl
8mM Urea	3M	37.5 µl
2M KOH	0.1M	5 µl
50mM EDTA	1mM	2 µl

1. Incubate at 90°C for 10 minutes in the above buffer.
2. Neutralize and EtOH precipitate DNA using:

	1x
X-link reversed sample	100 µl
ddH2O	200 µl
3M NaOAc pH 5.2	45 µl
2 µl GlycoBlue	2 µl
100% EtOH	1 ml

3. Incubate at -20°C for 1 hour.
4. Centrifuge at 4°C max speed for 30 min. Discard supernatant after centrifuge.
5. Rinse the pellet with 750 ul 70% EtOH.
6. Centrifuge at 4°C max speed for 5 min. Discard supernatant after centrifuge.
7. Air dry pellet for ~5 min at RT.
8. Resuspend each pellet in 50 µl ddH2O.
9. Perform pilot PCR to determine optimal number of amplification cycles:

Sample	10 µl (20% of total)
5x HF Buffer	20 µl
10 mM dNTP mix	2 µl
100 µM P5 oligo	0.4 µl
100 µM P7 oligo	0.4 µl
Phusion Poly (homemade)	1 µl
ddH2O	66.2 µl

bioRxiv preprint doi: https://doi.org/10.1101/2023.12.04.569951; this version posted December 6, 2023. The copyright holder for this preprint (which was not certified by peer review) is the author/funder, who has granted bioRxiv a license to display the preprint in perpetuity. It is made available under aCC-BY 4.0 International license.

10. Divide each 100 µl PCR reaction into 4x 25 µl aliquots. Run the following PCR with varying cycle numbers (14, 18, 22, 26 cycles). 95°C 5minutes, (95°C 20sec, 58°C 20sec, 72°C 30sec - 14, 18, 22, 26x, 72°C 5min, 4°C ∞
11. Run 12 µl (half) of each PCR reaction on 1.2% Agarose gel and determine the appropriate PCR cycle for the final library amplification.

12. Perform Final PCR amplification for library prep:

Sample	40 µl (80% of total)
5x HF Buffer	80 µl
10 mM dNTP mix	8 µl
100 µM P5 oligo	1.6 µl
100 µM P7 oligo	1.6 µl
Phusion Poly (homemade)	4 µl
ddH2O	264.8 µl

13. Divide each 400 ±l PCR reaction into 4x 100 µl aliquots. Run the following PCR with predetermined number of cycles.
14. After PCR, EtOH precipitate PCR products to reduce sample volume for AmPure bead clean-up.
15. Resuspend each sample with 100 µl ddH2O.
16. Add 160 µl AmPure bead slurry (1.6X) to each sample. Incubate at RT for 5 min on rotator (or Thermomixer 600 rpm).
17. Collect beads on a magnet. Discard supernatant.
18. Wash bead pellet 2x with 200 µl 75% EtOH without disturbing the pellet. Discard the wash
19. Air dry beads ~5min at RT.
20. Elute DNA with 20 µl of 10 mM Tris-Cl pH 8.5 (Qiagen EB buffer).
21. Quantitate DNA concentration using 2 µl samples with Qubit HS dsDNA Assay.
22. Submit samples for Illumina sequencing (ideally paired-end).

bioRxiv preprint doi: https://doi.org/10.1101/2023.12.04.569951; this version posted December 6, 2023. The copyright holder for this preprint (which was not certified by peer review) is the author/funder, who has granted bioRxiv a license to display the preprint in perpetuity. It is made available under aCC-BY 4.0 International license.

Femto-seq on cells fixed using DNA-FISH protocols.

Metaphase DNA-FISH. DNA-FISH was performed using commercially available FISH PAINT probes from KromaTiD (Longmont, CO) and performed using their standardized protocol. In short, cells were arrested in metaphase with 10 µl of 10 µg/ml colchicine and cells harvested after 4 hrs. After swelling using 75 mM KCl hypotonic solution, suspensions were fixed in 3:1 methanol and acetic acid. 20-30 µl of cell suspension was dropped onto glass slides and allowed to dry as per standard metaphase slide preparation[7]. Widefield images were taken using a 63x oil 1.4 NA objective on an inverted Nikon eclipse TI fluorescence microscope.

DNA-FISH Streptavidin Staining. Alexa-647-Streptavidin staining was performed on Femto-seq photobiotinylated samples to demonstrate the method also works in fixed samples. Samples were both fixed using a 4%PF 3D FISH protocol and 3:1 methanol acetic acid fixation for metaphase preparations. Samples prepared for metaphase FISH were prepared following the manufacturer's protocol (KromaTiD) as described above. Samples prepared for 3D FISH were prepared following standard protocols[8]. Both types of fixed samples were washed in PBS, then incubated in 200 µM AP3B in PBS for 1 hour at 37°C and immediately used for site specific photobiotinylation. Following photobiotinylation, cells were washed with PBS for 1 hr at 37°C. Then 1 ml of 1:500 dilution of 1 mg/ml Alexa-647-streptavidin was incubated with the cells for 30 minutes. Cells were then washed with PBS for 30 minutes and stained with DAPI mounting solution and imaged.

bioRxiv preprint doi: https://doi.org/10.1101/2023.12.04.569951; this version posted December 6, 2023. The copyright holder for this preprint (which was not certified by peer review) is the author/funder, who has granted bioRxiv a license to display the preprint in perpetuity. It is made available under aCC-BY 4.0 International license.

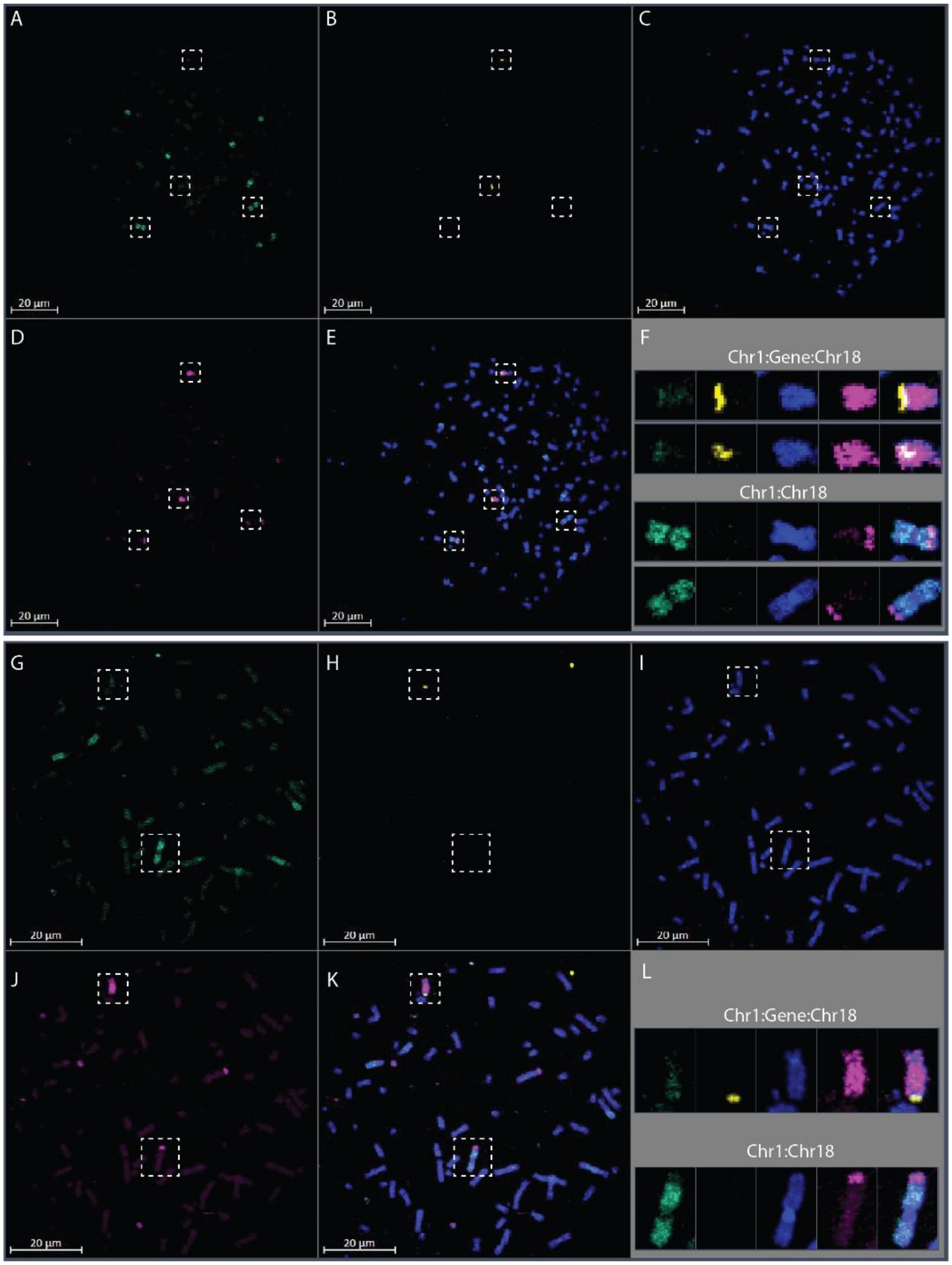

Figure S2. Representative Metaphase Spreads. **A-E** Metaphase DNA-FISH on U2OS cells. LacO locus in yellow, Chr1 in cyan, Chr18 in magenta, DAPI in blue. Boxes around the selected chromosomes show the chr1: chr18 translocation and new location of the YFP locus, **F** Enlarged view of the selected chromosomes showing the translocation of the YFP locus to Chr18. **G-K** Metaphase DNA-FISH on U2OS cells. LacO locus in yellow, Chr1 in cyan, Chr18 in magenta, DAPI in blue. Boxes around the selected chromosomes show the chr1: chr18 translocation and new location of the YFP locus. **L** Enlarged view of the selected chromosomes showing the translocation of the YFP locus to Chr18

bioRxiv preprint doi: https://doi.org/10.1101/2023.12.04.569951; this version posted December 6, 2023. The copyright holder for this preprint (which was not certified by peer review) is the author/funder, who has granted bioRxiv a license to display the preprint in perpetuity. It is made available under aCC-BY 4.0 International license.

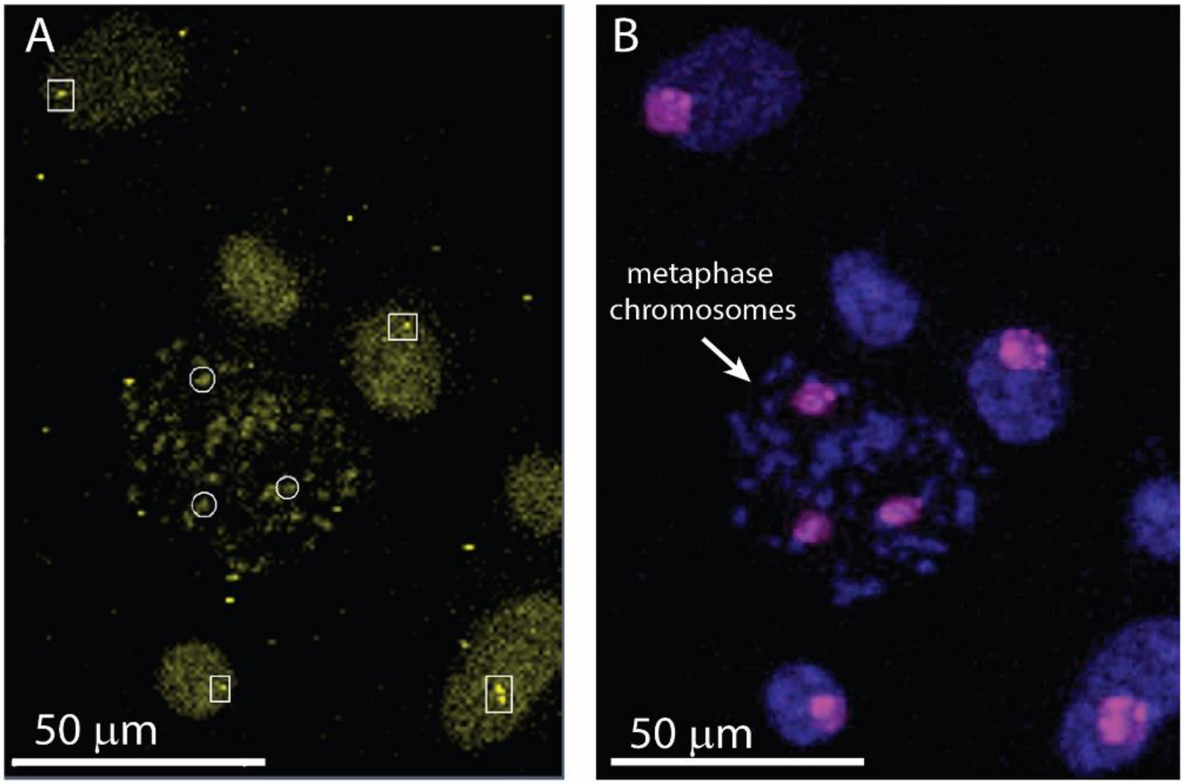

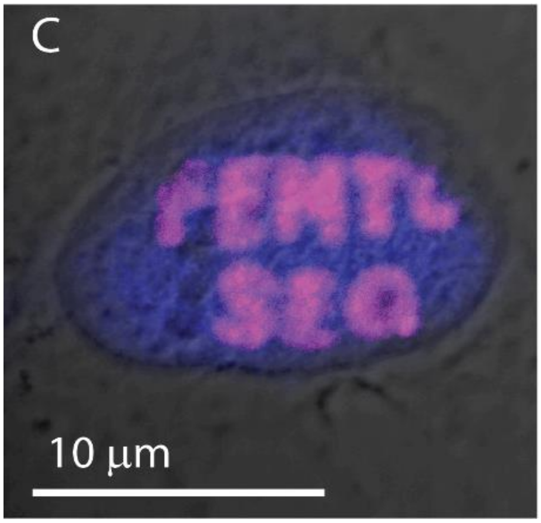

D

Treat-ment	Starting Amount	After Streptavidin Pull- Down	After Crosslink Reversal and DNA Purification
No PFA Fixation	100 ng	73 ng	18 ng
PFA Fixed 1	100 ng	71 ng	16 ng
PFA Fixed 2	100 ng	69 ng	64 ng

Figure S3. Demonstration of AP3B photobiotinylation in fixed cells. A. DNA-FISH to the YFP locus in cells fixed in Carnoy's fixative (3:1 methanol and acetic acid) for metaphase spread, white boxes show the areas for localized biotinylation using the 700 nm two-photon targeting. **B**. Alexa-647-Streptavidin (Magenta) and DAPI (Blue) labeling after photobiotinylation showing that two-photon mediated AP3B biotinylation can be used to in both cells and metaphase chromosomes in Carnoy's fixed cells. **C.** Representative photobiotinylation in 4% Paraformaldehyde fixed cells prepared for 3D-FISH protocol using a standard 3D-FISH protocol[4]. **D.** DNA recovery amounts for both non-fixed cells and fixed in 4% PFA cells under untargeted UV box photo-biotinylation. PFA-Fixed 1 and Non-Fixed used the standard ethanol precipitation for DNA purification after crosslink reversal, while PFA-Fixed 2 used a DNA purification column (Zymo Research DNA Clean and Concentrator).

bioRxiv preprint doi: https://doi.org/10.1101/2023.12.04.569951; this version posted December 6, 2023. The copyright holder for this preprint (which was not certified by peer review) is the author/funder, who has granted bioRxiv a license to display the preprint in perpetuity. It is made available under aCC-BY 4.0 International license.

References

1. Wielenberg K, Wang M, Yang M, Ozer A, Lis JT, Lin H. An improved 4′-aminomethyltrioxsalen-based nucleic acid crosslinker for biotinylation of double-stranded DNA or RNA. RSC Advances. 2020;10(65):39870-4. doi: 10.1039/D0RA07437C.
2. Zhao, R., et al., Gene bookmarking accelerates the kinetics of post-mitotic transcriptional re-activation. Nature Cell Biology, 2011. 13(11): p. 1295-1304
3. Singh A, Van Slyke AL, Sirenko M, Song A, Kammermeier PJ, Zipfel WR. Stoichiometric analysis of protein complexes by cell fusion and single molecule imaging. Sci Rep. 2020 Sep 10;10(1):14866. doi: 10.1038/s41598-020-71630-6. PMID: 32913201; PMCID: PMC7483473.
4. Langmead B, Salzberg S. Fast gapped-read alignment with Bowtie 2. Nature Methods. 2012, 9:357-359.
5. BEDTools: a flexible suite of utilities for comparing genomic features. Aaron R. Quinlan, Ira M. Hall, Bioinformatics, Volume 26, Issue 6, March 2010, Pages 841-842.
6. Ramírez, Fidel, Devon P. Ryan, Björn Grüning, Vivek Bhardwaj, Fabian Kilpert, Andreas S. Richter, Steffen Heyne, Friederike Dündar, and Thomas Manke. deepTools2: A next Generation Web Server for Deep-Sequencing Data Analysis. Nucleic Acids Research (2016). doi:10.1093/nar/gkw257.
7. "Fish Slide Making Protocol for Metaphase Spreads." Creative Bioarray, www.creative-bioarray.com/protocol/fish-slide-making-protocol-for-metaphase-spreads.htm.
8. Bolland DJ, King MR, Reik W, Corcoran AE, Krueger C. Robust 3D DNA FISH using directly labeled probes. J Vis Exp. 2013 Aug 15;(78):50587. doi: 10.3791/50587. PMID: 23978815; PMCID: PMC3846859.
